# Exploring the potential of metaphase I oocytes unresponsive to rescue IVM: fertilization, embryo development, and pregnancy outcomes

**DOI:** 10.1007/s00404-026-08484-2

**Published:** 2026-07-18

**Authors:** Ayşe Altun Akpınar, Burçin Karamustafaoğlu Balcı, Cigdem Celik, Ömer Demir, Cemil Akgül

**Affiliations:** 1 Department of Obstetrics and Gynecology, Division of Reproductive Endocrinology and Infertility, Faculty of Medicine, Istanbul University, Capa, Fatih, Istanbul, Turkey; 2 Department of Obstetrics and Gynecology, Division of Reproductive Endocrinology and Infertility, Faculty of Medicine, Karadeniz Technical University, Trabzon, Turkey

**Keywords:** Immature oocytes, Intracytoplasmic sperm injection (ICSI), In vitro maturation (IVM)

## Abstract

**Background:**

The aim of this prospective cohort study was to evaluate the developmental potential of metaphase I (MI) oocytes that failed to mature following rescue in vitro maturation (IVM), and to investigate the roles of artificial oocyte activation (AOA) and meiotic spindle presence in fertilization and early embryo development.

**Methods:**

A total of 49 patients were included in the analysis. The study population was divided into 2 groups: an AOA group (28 patients, 42 MI oocytes) undergoing intracytoplasmic sperm injection (ICSI) followed by calcium ionophore-mediated activation, and a non-AOA control group (21 patients, 40 MI oocytes) undergoing ICSI without activation. Fertilization, embryo development, and clinical outcomes were compared between the groups. In addition, meiotic spindle presence was assessed using polarized light microscopy, and patients were categorized as spindle-positive or spindle-negative.

**Results:**

Fertilization occurred in 23 of 42 MI oocytes (54.7%) in the AOA group, compared with 1 of 40 (2.5%) in the non-AOA group (*p* < 0.001). In the control group, fertilization was observed only in a spindle-positive patient, and the resulting embryo arrested before transfer. In contrast, embryos derived from AOA-treated oocytes were able to progress to the transfer stage. Among AOA-treated patients, fertilization rates were higher in the spindle-positive group than in the spindle-negative group (71.4% vs. 50.0%), although this difference did not reach statistical significance. A total of eight embryos reached the transfer stage, resulting in one biochemical pregnancy and one clinical pregnancy leading to a live birth.

**Conclusion:**

MI oocytes that fail to mature after rescue IVM appear to retain limited developmental potential. The use of artificial oocyte activation was associated with higher fertilization rates and enabled embryo development to progress to transferable stages in this cohort. The presence of a meiotic spindle may be associated with improved developmental competence and could represent a potential non-invasive marker; however, this observation requires further validation. Given the limited sample size and exploratory nature of the study, these findings should be interpreted with caution, and larger studies are needed to confirm their clinical relevance.

**Supplementary Information:**

The online version contains supplementary material available at https://doi.org/10.1007/s00404-026-08484-2.

## What does this study add to the clinical work

**Table:** 

Although MI oocytes that fail to mature after rescue IVM have limited developmental potential, some may still contribute to embryo transfer and pregnancy. Meiotic spindle visualization may serve as a useful non-invasive marker for selecting MI oocytes with higher developmental competence.	

## Introduction

In assisted reproductive techniques (ART), although multiple oocytes are retrieved following ovarian stimulation, only a small proportion ultimately result in live birth due to sequential biological elimination and clinical selection during fertilization, embryo development, and implantation stages [1]. The maturity of the oocyte plays a critical role in the success of IVF treatments. For optimal fertilization and embryo development, microinjection is typically performed on metaphase II (MII) oocytes. However, studies indicate that a substantial proportion of retrieved oocytes—approximately 15–25%—are immature at the time of retrieval [2, 3]. Ahmad et al. reported that immature oocytes can be subjected to in vitro maturation protocols to achieve MII status [2], while Shani et al. demonstrated that MII oocytes derived from initially immature oocytes retain the capacity to undergo normal fertilization and early embryonic development [3], supporting their potential use in clinical IVF practice.

Immature oocytes are classified into three stages of nuclear maturation: prophase I (PI), metaphase I (MI), and, less commonly, telophase I (TI). Oocytes arrested in PI, commonly referred to as germinal vesicle (GV) oocytes, have a large nucleus centrally located within the cytoplasm. The GV stage represents the earliest stage of maturation, and GV oocytes are almost always discarded during IVF-ICSI cycles. MI oocytes have progressed further through meiosis and are closer to maturity but require extrusion of the first polar body to transition into MII—the highest maturity stage an oocyte can achieve. TI oocytes, which represent a very brief transitional stage characterized by extrusion of the first polar body, are rarely distinguished in clinical practice and are typically classified as MI or early MII oocytes during ICSI.

Rescue in vitro maturation (IVM) is a technique used in IVF treatment in which immature oocytes, primarily MI oocytes, are cultured to achieve maturity for ICSI. Although in vitro-matured oocytes typically yield poorer outcomes compared with in vivo-matured oocytes [4], rescue IVM represents an important alternative for specific patient groups, such as those with polycystic ovary syndrome (PCOS), unsynchronized follicular cohorts, or poor ovarian response [5]. However, many MI oocytes do not respond to the IVM process, failing to extrude a polar body, complete nuclear maturation, or progress to the MII stage.

Conventional in vitro maturation involves the retrieval of immature oocytes from unstimulated or minimally stimulated ovaries, followed by intentional in vitro maturation prior to fertilization. This approach is primarily used to avoid ovarian hyperstimulation and to obtain mature oocytes from patients in whom immature oocytes are the intended target [6]. In contrast, rescue IVM refers to the in vitro maturation of immature oocytes (GV or MI) that are incidentally retrieved during controlled ovarian stimulation cycles aimed at collecting mature MII oocytes. In this setting, immature oocytes are not the primary objective but represent a secondary cohort. Rescue IVM is, therefore, performed to maximize oocyte utilization within conventional ART cycles [7]. Importantly, oocytes in stimulated cycles have already been exposed to supraphysiological gonadotropin levels, which may influence their nuclear–cytoplasmic maturation dynamics differently from those of oocytes retrieved in conventional IVM cycles [8].

ART clinics strive to optimize the number of embryos available for transfer and pregnancy. Despite advances in culture techniques, not all MI oocytes develop into MII oocytes. However, should MI oocytes always be discarded? This study investigates the clinical utility of MI oocytes for ICSI by evaluating their developmental competence when they fail to respond to rescue IVM procedures.

## Materials and methods

### Study description

This prospective cohort study was conducted at the Istanbul University, Istanbul Faculty of Medicine, Department of Obstetrics and Gynecology, IVF Unit. Ethical approval was obtained from the institutional ethics committee (approval no: E-54073746-108.99-2511084). Written informed consent was obtained from all participating couples.

Clinical and laboratory data from patients who underwent IVF-ICSI treatment at the unit between October 2023 and April 2024 were prospectively collected during the treatment process and subsequently analyzed. In accordance with national regulations, all IVF/ICSI cycles were performed using autologous oocytes. Patients who met the predefined inclusion criteria were included, whereas those meeting any exclusion criteria were excluded from the analysis.

### Inclusion criteria

- IVF cycles using autologous oocytes
- Women aged 18–40 years
- Body mass index (BMI) between 18.5 and 30 kg/m^2^
- Presence of at least one metaphase I (MI) oocyte that failed to progress to metaphase II (MII) despite rescue in vitro maturation (IVM) and was subsequently subjected to intracytoplasmic sperm injection (ICSI) at the MI stage

### Exclusion criteria

- Oncofertility patients
- Oocyte cryopreservation cycles
- Fertility preservation cycles
- Cycles in which all retrieved oocytes were at the MII stage
- Cycles with complete rescue IVM success, defined as all MI oocytes maturing to MII
- Cycles involving only germinal vesicle (GV) oocytes

The study population was divided into two groups based on the application of artificial oocyte activation. The decision to perform artificial oocyte activation (AOA) was made individually during the IVF-ICSI cycle in patients considered to have a high risk of fertilization failure or oocyte activation deficiency based on previous fertilization history, poor oocyte quality, or embryological assessment of the retrieved MI oocytes. The decision was made jointly by the treating clinicians and embryologists and was not randomized. Patients who underwent ICSI without subsequent AOA constituted the non-AOA control group. Therefore, the possibility of selection bias should be considered when interpreting comparisons between the groups.

The AOA group consisted of 28 patients with a total of 42 MI oocytes undergoing ICSI followed by calcium ionophore-mediated activation, whereas the non-AOA control group consisted of 21 patients with 40 MI oocytes undergoing ICSI without activation. Both groups were processed under identical laboratory conditions. Fertilization, cleavage, embryo development, and transfer outcomes were compared to evaluate the independent effect of artificial oocyte activation.

### Stimulation protocol

All patients underwent a GnRH antagonist protocol. Controlled ovarian hyperstimulation (COH) was initiated on day 3 of the menstrual cycle with recombinant FSH (Gonal-F, EMD Serono, MA, USA) at a daily dose of 75–300 IU. Pituitary suppression was achieved using cetrorelix 0.25 mg/day (Cetrotide, Serono, Geneva, Switzerland), initiated when at least one follicle reached a diameter of ≥ 14 mm. Final oocyte maturation was triggered with 6500 IU of hCG (Ovitrelle, Merck KGaA, Darmstadt, Germany) when at least two follicles reached a diameter of ≥ 17 mm.

### Oocyte retrieval (OPU)

Oocyte retrieval was performed 35–36 h after hCG administration using a Labotect device (Model 4014, Germany) and double-lumen OPU needles (Swemed 14,105, Sweden). Each follicle was meticulously aspirated into sterile tubes (Falcon 2003, USA/France) containing follicular fluid supplemented with GMOPS solution and GMOPS + HSA medium (Vitrolife, Sweden, EU). The aspirated contents were examined under a stereomicroscope (Olympus SZH10, Japan) in the embryology laboratory.

Oocytes were denuded approximately 3 h after retrieval and subsequently examined under an inverted microscope to assess the stage of nuclear maturation. Oocytes were classified into the following stages: metaphase II (MII), metaphase I (MI), germinal vesicle (GV), and grade IV (GIV).

GV oocytes represent the earliest stage of nuclear maturation and are characterized by the presence of a visible nucleus within the cytoplasm and the absence of meiotic progression.

GIV oocytes were defined as morphologically poor-quality MII oocytes exhibiting severe cytoplasmic and/or extracytoplasmic abnormalities, including dark or granular cytoplasm, large vacuoles, cytoplasmic inclusions, irregular oolemma, and zona pellucida abnormalities. These oocytes are considered to have compromised developmental competence and are typically associated with reduced fertilization and embryo development potential [9].

### Rescue IVM protocol

During the study period, rescue in vitro maturation (IVM) was performed in all cycles involving MI oocytes. Oocytes retrieved 36 h after hCG triggering were initially incubated for approximately 3 h under controlled atmospheric conditions (7% CO₂ and 5% O₂).

Following this incubation period, oocytes were denuded using hyaluronidase and mechanical pipetting techniques to remove the surrounding cumulus cells. Immature MI oocytes were subsequently reassessed at 40 h post-trigger.

Oocytes that remained at the MI stage were further cultured in LifeGlobal Total for Fertilization Medium (Cooper Surgical, Denmark) under the same atmospheric conditions (7% CO₂ and 5% O₂). This single-step culture medium contains essential ions, energy substrates (glucose, pyruvate, and lactate), amino acids, vitamins, and a defined protein source, and is optimized to support oocyte metabolism and promote maturation. It provides a stable environment for the short-term culture of immature oocytes without the need for sequential medium changes.

Intracytoplasmic sperm injection (ICSI) was performed 4 h after denudation on oocytes that remained at the MI stage. In this study, rescue IVM was implemented as a short-term culture protocol, with MI oocytes cultured for 4 h prior to ICSI.

### ICSI procedure protocol

Following denudation, oocytes were assessed for nuclear maturity under an inverted microscope. Metaphase II (MII) oocytes underwent intracytoplasmic sperm injection (ICSI) at approximately 40 h post-trigger using partner sperm.

Oocytes that remained at the immature metaphase I (MI) stage at approximately 40 h post-trigger were cultured for an additional 4 h. At approximately 44 h post-trigger, ICSI was performed on these oocytes, including those that had matured to MII during extended incubation as well as those that remained at the MI stage.

In cases of suspected maturation deficiency, artificial oocyte activation (AOA) using calcium ionophore has been proposed to enhance cytoplasmic maturation by inducing calcium oscillations, which are essential for meiotic resumption, oocyte activation, and early embryonic development, even in the absence of complete nuclear maturation.

In this study, MI oocytes were subjected to chemical activation using calcium ionophore (GM508 Cult-Active Calcium Ionophore, Germany) prepared in 30 µL droplets. The droplets were pre-incubated for at least 4 h at 37 °C under controlled conditions (pH 7.2–7.4; 5–7% CO₂).

Immediately following ICSI, MI oocytes were exposed to calcium ionophore-containing medium for 20 min to induce activation. Although the standard exposure duration for Cult-Active is commonly reported as 15 min [10], previous studies using calcimycin have demonstrated the safety of exposure durations of 20 min or longer [11]. Therefore, a 20-min exposure time was applied in this study.

Following activation, oocytes were washed in culture medium free of HEPES and MOPS, transferred to IVF culture medium (Vitrolife, Sweden, EU), and incubated until fertilization assessment.

### Assessment of meiotic spindle by polarized light microscopy

In addition to routine assessment of nuclear maturation, polarized light microscopy was used to evaluate the presence, visibility, and localization of the meiotic spindle in MI oocytes that failed to mature following rescue IVM. All oocytes were examined using a PolScope system integrated into an inverted microscope (Nikon Eclipse Ti-U, Tokyo, Japan).

Oocytes were placed in 20–30 µL microdroplets of culture medium under mineral oil and maintained at 37 °C during imaging. Each oocyte was evaluated within 5–10 min to minimize thermal and pH-related stress. Spindle visualization was performed using standardized exposure, contrast, and birefringence settings across all samples. A spindle was classified as visible when a birefringent, barrel-shaped microtubule structure with a clearly defined longitudinal axis was identified.

MI oocytes were categorized as follows:

- *MI–no spindle* No birefringent structure was detected, and no polar body was present.
- *MI–spindle-positive (transition MI)* A meiotic spindle was detected despite the absence of the first polar body

Spindle visibility rates and morphological characteristics of MI oocytes were included in the analysis to determine whether MI oocytes failing IVM exhibited signs of ongoing nuclear progression toward the MII stage.

### Fertilization assessment

Fertilization was assessed approximately 16–20 h after intracytoplasmic sperm injection (ICSI) using an inverted microscope equipped with Hoffman modulation contrast optics. Normal fertilization was defined by the presence of two distinct pronuclei (2PN) and two polar bodies (2 PB).

Abnormal fertilization patterns were also recorded, including:

- 0PN (absence of pronuclei)
- 1PN (single pronucleus)
-  ≥ 3PN (polypronuclear zygotes)
- Oocyte degeneration

Pronuclear morphology was evaluated according to established morphological criteria, including:

- Size and symmetry of pronuclei
- Alignment and distribution of nucleolar precursor bodies (NPBs)
- Cytoplasmic appearance

Zygotes were morphologically graded as follows:

- Zygote I (ZI): Very good
- Zygote II (ZII): Good
- Zygote III (ZIII): Moderate
- Zygote IV (ZIV): Poor

However, zygote grading was not used for outcome comparisons, and fertilization outcomes were reported solely as fertilization rates based on 2PN formation. Only normally fertilized oocytes (2PN) were included in subsequent embryo culture and developmental analyses.

Fertilization and zygote assessment were performed in accordance with the consensus criteria established by the Alpha Scientists in Reproductive Medicine and the ESHRE Special Interest Group of Embryology (Istanbul consensus on embryo assessment).

### Embryo development

Embryo development was assessed at the cleavage stage (day 2–4), depending on the individualized clinical transfer strategy. Cleavage-stage embryos were graded according to standard morphological criteria, including blastomere number, symmetry, and degree of fragmentation.

To enable standardized comparisons across patients with different transfer timings, embryos were further classified into three overall developmental quality categories (good, moderate, and poor) based on their stage-appropriate morphological grade, irrespective of the day of development. This day-independent classification was used for statistical analysis.

The cleavage stage refers to the period from the 2-cell stage (day 1 post-oocyte retrieval) to the morula stage, characterized by 15–20 compacted cells (day 4 post-oocyte retrieval). During this period, the embryo undergoes sequential mitotic divisions, resulting in an increase in cell number.

Cleavage-stage embryo quality was defined as follows:

- *Grade I* Blastomeres equal in size, with no or minimal fragmentation (0–5%)
- *Grade II* Blastomeres equal or slightly unequal in size, with 5–10% fragmentation
- *Grade III* Blastomeres unequal in size, with 10–25% fragmentation
- *Grade IV* Blastomeres markedly unequal in size, with > 25% fragmentation

Embryo assessment was performed in accordance with the consensus criteria established by the Alpha Scientists in Reproductive Medicine and the ESHRE Special Interest Group of Embryology (Istanbul consensus on embryo assessment).

### Embryo transfer

Embryo development was monitored daily, beginning with fertilization assessment and subsequent cleavage-stage evaluations from day 1. Selected embryos were transferred according to individualized clinical protocols.

Pregnancy was assessed approximately 12 days after embryo transfer by measuring serum beta-human chorionic gonadotropin (β-hCG) levels. Clinical follow-up was initiated in patients with positive β-hCG results.

All embryo transfers were performed under ultrasound guidance by the same infertility and reproductive endocrinology specialist. Luteal phase support was administered using intramuscular progesterone in oil and vaginal progesterone supplementation.

The decision to perform a freeze-all strategy was based on individual patient characteristics, including endometrial status, hormone levels, and the risk of ovarian hyperstimulation syndrome (OHSS).

### Outcome measures

Reproductive outcomes were defined as follows:

- *Implantation rate* A positive serum beta-human chorionic gonadotropin (β-hCG) level measured 14 days after embryo transfer
- *Clinical pregnancy* The presence of one or more intrauterine gestational sacs detected by ultrasound at 7 weeks of gestation
- *Ongoing pregnancy* A pregnancy confirmed by ultrasound to have progressed beyond 12 weeks of gestation

### Statistical analysis

Statistical analyses were performed using IBM SPSS Statistics (IBM Corp., Armonk, NY, USA). The normality of data distribution was assessed using the Shapiro–Wilk test.

Continuous variables were expressed as mean ± standard deviation (SD) or median (minimum–maximum), depending on data distribution. Categorical variables were presented as frequencies (*n*) and percentages (%).

Comparisons between groups were performed using the independent samples *t* test for normally distributed continuous variables and the Mann–Whitney *U* test for non-normally distributed variables. The Chi-square test or Fisher’s exact test was used for categorical variables, as appropriate. A *p* value of < 0.05 was considered statistically significant.

A post hoc power analysis was performed for the primary outcome (fertilization rate) using the observed proportions in the AOA and non-AOA groups (54.7% vs. 2.5%). With a two-sided alpha level of 0.05, the achieved statistical power was calculated to be > 99%, indicating adequate power for the primary endpoint. Given the limited sample size, all secondary endpoints, including embryo development, spindle assessment, and pregnancy outcomes, were considered exploratory analyses and interpreted cautiously.

A STROBE checklist and patient flow diagram are provided as Supplementary Materials (Supplementary File 1, Supplementary Fig. 1).

## Results

During the study period, 263 patients underwent 276 ICSI cycles in our clinic. A total of 49 patients were included in the analysis, comprising 28 patients in the AOA group and 21 patients in the non-AOA control group. The mean age of the patients was 36.4 ± 4 years in the AOA group and 37.1 ± 4.2 years in the control group (*p* = 0.48). The mean partner age was 38.5 ± 3.9 and 36.4 ± 3.3 years, respectively (*p* = 0.09).

The primary indication for treatment in both groups was low ovarian reserve (39.3% vs. 42.8%). Other indications, including male factor, endometriosis, tubal factor, unexplained infertility, and combined male factor with low ovarian reserve, were similarly distributed between the groups.

Baseline clinical and hormonal parameters, including body mass index, infertility duration, antral follicle count, AMH, FSH, LH, and estradiol levels, were comparable between the two groups. The mean number of retrieved oocytes was 6.7 ± 4.7 in the AOA group and 4.8 ± 3.6 in the control group. The distribution of oocyte maturity stages (MII, MI, GV, and GIV) was also similar between groups.

Overall, no statistically significant differences were observed between the groups in terms of baseline demographic, clinical, and laboratory characteristics (all *p* > 0.05) (Table 1).

**Table 1:** Baseline demographic data, hormone levels, and reproductive characteristics of the study population. Data are presented as mean ± standard deviation (range: min–max). There were no statistically significant differences between the groups in terms of baseline characteristics (all *p* > 0.05)

	AOA group (*n* = 28)	Non-AOA group (*n* = 21)	*p* value
Indication	Low ovarian reserve (LOR) – 11 (%39.3) Male factor – 6 (%21.4) Endometriosis– 3 (%10.7) Tubal factor– 3 (%10.7) Unexplained– 3 (%10.7) Male factor + LOR – 2 (%7.1)	Low ovarian reserve (LOR) – 9 (%42.8) Male factor – 5 (%23.8) Endometriosis– 1 (% 4.7) Tubal factor– 1 (% 4.7) Unexplained– 2 (%9) Male factor + LOR – 3 (%14)	0.82
Age (patient)	36.4 ± 4 (27 – 44)	37.1 ± 4.2 (26 – 43)	0.48
Age (partner)	38.5 ± 3.9 (31 – 47)	36.4 ± 3.3 (29 – 44)	0.09
Body mass index	26 (19 – 39)	24.2 (16.4 – 31.3)	0.21
Infertility period (year)	4 (0.25 – 14)	4.6 (0.50 – 13)	0.67
Antral follicle count	6 (1 – 13)	7 (2 – 13)	0.55
Anti-Mullerian hormone	2.1 ± 1.9 (0.2 – 9.3)	2.5 ± 1.2 (0.24 – 8.3)	0.39
Follicle-stimulating hormone	7.2 ± 2.9 (0.3 – 13.7)	7.6 ± 3.4 (0.34 – 12.5)	0.62
Luteinizing hormone	6.1 ± 3 (0.3 – 14)	6.8 ± 3 (0.36 – 13.8)	0.48
Estradiol	56.9 ± 25.2 (16 – 153)	62.5 ± 26.4 (15 – 142)	0.41
Number of oocytes	6.7 ± 4.7 (1 – 21)	4.8 ± 3.6 (2 – 16)	0.12
MII oocytes	4 (1 – 18)	5 (1 – 13)	0.33
MI oocytes	2 (1 – 5)	2 (1 – 4)	0.91
GV oocytes	1 (0 – 4)	1 (0 – 3)	0.88
GIV oocytes	2 (0 – 7)	2 (0 – 5)	0.95

**Fig. 1:**
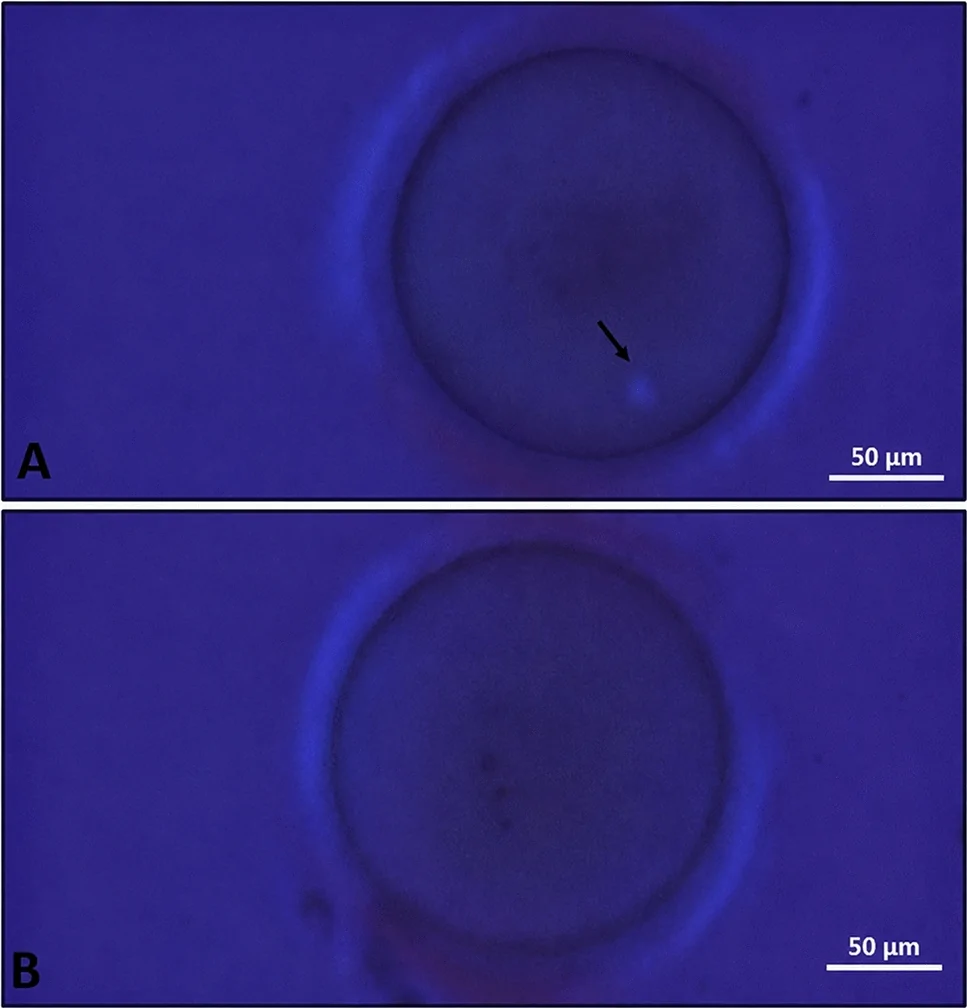
Polarized light microscopy images of MI oocytes resistant to rescue IVM. **A** MI–Spindle Positive: The oocyte shows a clearly detectable located meiotic spindle (arrow) despite the absence of the first polar body, indicating a transitional late-MI stage. B. MI–No Spindle: No birefringent spindle structure is observed and no polar body is present, consistent with an early MI stage. Imaged using a 40X objective (final magnification ~400X).

Polarized light microscopy was successfully performed on MI oocytes resistant to rescue IVM. Two spindle patterns were identified. A subset of MI oocytes displayed a located metaphase spindle despite the absence of the first polar body (MI–Spindle Positive), indicating ongoing nuclear maturation without completed cytokinesis. In contrast, classic MI oocytes showed no birefringent structure (MI–No Spindle). These findings suggest that a proportion of MI oocytes failing short-term IVM may nevertheless enter late-MI/transition stages, potentially retaining partial meiotic competence. Representative polarized images of spindle-positive and spindle-negative MI oocytes are provided in Fig. 1.

A total of 42 MI oocytes underwent ICSI followed by calcium ionophore activation, of which 23 were fertilized, yielding a fertilization rate of 54.7%. Among the 23 fertilized zygotes, 7 (30.4%) were classified as ZII, 11 (47.8%) as ZIII, and 5 (21.7%) as ZIV (Table 2, Table 3). A control group of 40 MI oocytes that did not undergo AOA was analyzed for comparison. In this group, only one oocyte (2.5%) was fertilized, which developed into a Day 2 Grade I embryo but subsequently arrested and did not reach embryo transfer. Fertilization rates were significantly higher in the AOA group compared to the non-AOA group (54.7% vs. 2.5%, *p* < 0.001) (Table 2). No embryo transfer was performed in the non-AOA group.

**Table 2:** Comparison of fertilization and embryo development outcomes between AOA-treated and non-AOA MI oocytes. Statistical comparison beyond fertilization was not performed due to the limited sample size in the non-AOA group

Outcome	AOA ( +) MI oocytes (*n* = 42)	AOA (–) MI oocytes (*n* = 40)	*p* value
Fertilization rate	23/42 (54.7%)	1/40 (2.5%)	< 0.001
Cleavage embryos	11/23 (47.8%)	1/1 (100%)	NA
High-quality embryos	7/23 (30.4%)	1/1 (100%)	NA
Embryos reaching transfer	5/23 (21.7%)	0/1 (0%)	NA
Embryo arrest	3/23 (13.0%)	1/1 (100%)	NA

**Table 3:** Embryological and clinical outcomes of MI oocytes following intracytoplasmic sperm injection (ICSI) combined with artificial oocyte activation (AOA)

Parameter	Number of oocytes, n/N (%)
Total MI oocytes injected	42
Fertilized oocytes (2PN)	23
Fertilization rate (mean ± SD)	54.7 ± 48.4 (0–100)

Pronuclear morphology	
Parameter	Number of zygotes (*n* = 23), n/N (%)
2PN-ZI	0
2PN-ZII	7/23(30.4%)
2PN-ZIII	11/23 (47.8%)
2PN-ZIV	5/23 (21.7%)

Embryo development	
Parameter	Number of patients (*n* = 17), n/N (%)
*Day 2 embryos*	11/17 (64.7%)
D2 Grade I	7/17 (41.2%)
D2 Grade II	3/17 (17.6%)
D2 Grade III	1/17 (5.9%)
*Day 3 embryos*	11/17 (64.7%)
D3 Grade I	4/17 (23.5%)
D3 Grade II	5/17 (29.4%)
D3 Grade III	2/17 (11.8%)
Day 4 embryos	8/17 (47.1%)

Fresh embryo transfers	
Parameter	Number of patients (*n* = 14), n/N (%)
Fresh transfers performed	5/14 (35.7%)
Grade I embryos transferred	5/5 (100%)
Implantation rate (per transfer)	1/5 (20%)
Clinical pregnancy (per transfer)	1/5 (20%)
Negative (per transfer)	3/5 (60%)

Frozen embryo transfers	
Parameter	Number of patients (*n* = 3), n/N (%)
Frozen transfers	3/3 (100%)
Grade I embryos	3/3 (100%)
Clinical pregnancy	0
Negative	3/3 (100%)

Data are expressed as n/N (%). Fertilization outcomes are calculated per injected oocyte, whereas embryo development and clinical outcomes are evaluated on a patient basis. Only normally fertilized oocytes (2PN) were included in further developmental analysis.

In the AOA-treated group, on Day 2, 11 embryos (64.7%) had cleaved, of which 41.2% were classified as Grade I. All of these embryos were cultured to Day 3. By Day 3, two embryos that had been graded as Grade I on Day 2 were classified as Grade II, and one was classified as Grade III. Three embryos arrested during development, while 8 embryos (47.1%) continued to Day 4. Of these 8 embryos, 3 were frozen as part of freeze-all cycles, and 5 out of 14 embryos (35.7%) were fresh transferred. One of the five fresh embryo transfers resulted in pregnancy. The implantation rate was 20% based on the number of fresh embryos transferred (Table 3). This pregnancy resulted in a healthy live birth. Among the remaining three frozen embryos, all were thawed and transferred (single embryo transfer). Two patients did not achieve pregnancy and one patient experienced biochemical pregnancy. Therefore, among all eight transferred embryos, one biochemical pregnancy and one term live birth were observed (implantation rate 25%, live birth rate 12.5%).

### Outcomes according to meiotic spindle presence

In the AOA-treated group, a total of 28 patients were evaluated. Based on polarized light microscopy findings, patients were categorized into 2 groups: 14 patients with detectable meiotic spindle and 14 patients without detectable spindle.

In the non-AOA control group, 21 patients were evaluated, of whom 10 had detectable spindle and 11 had no detectable spindle.

#### Fertilization outcomes

In the AOA-treated group, the fertilization rate was higher in patients with spindle-positive findings compared to those without detectable spindle (71.4% [10/14] vs. 50.0% [7/14]). However, this difference did not reach statistical significance (χ^2^ = 1.35, *p* = 0.24). Fisher’s exact test also confirmed the absence of statistical significance (*p* = 0.27) (Table 4).

**Table 4:** Comparison of embryological and clinical outcomes according to meiotic spindle presence in the AOA-treated group

Parameter	Spindle-present number of patients (*n* = 14)	Spindle-absent number of patients (*n* = 14)	*p* value
Fertilization rate	10/14 (71.4%)	7/14 (50.0%)	0.24
Grade I embryos (fresh transfer)	4/5 (80%)	1/5 (20%)	—
Grade I embryos (frozen)	3/3 (100%)	0	—
Clinical pregnancy per fresh transfer	1/4 (25%)	0/1 (0%)	1.00
Positive pregnancy per fresh transfer	2/4 (50%)	0/1 (0%)	0.40
Frozen embryo pregnancy rate	0/3 (0%)	—	—

Data are expressed as n/N (%), calculated on a patient basis. Patients were categorized according to the presence or absence of a detectable meiotic spindle assessed by polarized light microscopy. Fertilization, embryo development, and clinical outcomes were compared between groups. Chi-square and Fisher’s exact tests were used as appropriate. Statistical comparisons were not performed for some parameters due to small sample size

In the non-AOA control group, only one fertilization was observed, and this occurred in a spindle-positive patient, while no fertilization was detected in spindle-negative patients.

#### Embryo quality and development

Among fertilized oocytes:

- All three cryopreserved Grade I embryos originated from the spindle-present group.
- Of the five fresh embryo transfers, four embryos (80%) were derived from spindle-positive oocytes and one embryo (20%) from the spindle-negative group.
- All transferred embryos were Grade I.

#### Pregnancy outcomes (fresh transfers)

Five fresh embryo transfers were performed:

- *Spindle-present group* 4 transfers.
- *Spindle-absent group* 1 transfer.

#### Clinical pregnancy

Clinical pregnancy occurred in 1 of 4 transfers (25%) in the spindle-present group and in 0 of 1 transfers (0%) in the spindle-absent group.

Fisher’s exact test revealed no statistical significance (*p* = 1.00).

#### Overall positive pregnancy (biochemical + clinical)

Positive pregnancy was observed in two of four transfers (50%) in the spindle-present group, whereas no pregnancies were observed in the spindle-absent group (0%).

Fisher’s exact test showed no statistically significant difference (*p* ≈ 0.40).

#### Frozen embryo transfers

Three frozen Grade I embryos (all from the spindle-present group) were transferred.

No biochemical or clinical pregnancies were observed (Table 4).

## Discussion

This study explored the developmental potential of MI oocytes that did not respond to rescue IVM. The findings suggest that, despite their well-known limitations compared with in vivo-matured MII oocytes, immature MI oocytes may still undergo fertilization, embryo development, and, in some cases, result in pregnancy. These results indicate that MI oocytes, which are usually discarded in routine IVF practice, could represent a potential additional resource for patients undergoing assisted reproduction.

A significant and unresolved issue in IVF-ICSI cycles is immature oocytes. In standard practice, insemination is performed exclusively on MII oocytes. While rates vary slightly between studies, approximately 4% of collected oocytes are in metaphase I, and 11% are at the germinal vesicle stage [12, 13–15]. Other studies have reported that around 20% of oocytes are immature [16]. Even though the clinical efficacy and safety of in vitro maturation in stimulated cycles are subjects of ongoing debate, rescue IVM is routinely applied to all MI oocytes, regardless of the patient’s MII oocyte count, as part of a strategy to potentially optimize cycle outcomes in our IVF Unit, because each mature oocyte may contribute to cumulative pregnancy and live birth rates [17]. Additionally, a higher proportion of immature oocytes within the total yield has been associated with reduced pregnancy potential of mature sibling MII oocytes [11]. For this reason, every MI oocyte is considered valuable. If MI oocytes extrude the first polar body within a few hours, they are rescued, matured in vitro, and subsequently inseminated. Approximately 60% of immature oocytes progress to the MII stage [17]. However, when IVM fails and MI oocytes do not mature, they are typically discarded in most IVF units.

The short-term IVM protocol applied in this study differs from conventional IVM. Unlike the traditional long-term IVM approach—where immature oocytes are cultured for approximately 20–24 h until they reach the MII stage, and ICSI is performed only after the completion of nuclear maturation—our protocol consisted of a limited 4-h culture period. Following this 4-h IVM period, nuclear maturation was assessed. MI oocytes that failed to progress to the MII stage were included in the study. This methodological distinction is clinically and biologically relevant. Conventional IVM aims to achieve full nuclear maturation prior to fertilization, with the assumption that cytoplasmic maturation occurs in parallel during extended culture []. In contrast, the short-term IVM strategy used in the present study provided only a brief culture window before proceeding with ICSI, even in the absence of polar body extrusion. Therefore, the developmental outcomes observed in our cohort reflect fertilization of MI-stage oocytes following short-term culture rather than embryos derived from fully matured MII oocytes obtained through standard IVM protocols. Interestingly, in the short-term IVM group, fertilization and clinical pregnancy following MI-stage ICSI were observed exclusively in oocytes in which a meiotic spindle was detectable under polarized light microscopy. This finding may provide insight into the biological differences between short-term and conventional long-term IVM approaches.

In long-term IVM protocols, oocytes are cultured until polar body extrusion is observed, and ICSI is performed only after apparent nuclear maturation to the MII stage. However, nuclear maturation alone does not always guarantee adequate cytoplasmic maturation or optimal spindle integrity. In contrast, our findings suggest that, in short-term IVM cycles, the presence of a visible meiotic spindle may serve as a more relevant indicator of functional maturation and developmental competence than time-dependent culture alone. This may imply that spindle visualization could help identify a subset of MI oocytes with intrinsic maturation potential, even in the absence of complete progression to MII within a conventional culture time frame. Therefore, while long-term IVM relies primarily on polar body extrusion as a maturation marker, short-term IVM outcomes in our study appear to be closely associated with spindle presence, highlighting a potentially different biological selection mechanism.

This study showed that MI oocytes that failed to mature following rescue IVM demonstrated measurable developmental potential, with observable fertilization and embryo formation rates. Polarized light microscopy revealed that MI oocytes are not a uniform subgroup. In a subset of MI oocytes, a well-defined metaphase spindle was observed despite the absence of the first polar body, suggesting that these oocytes had initiated nuclear maturation and may represent a “late MI/MI–MII transitional” stage. In contrast, spindle-negative MI oocytes reflected an earlier phase of meiosis. This biological variability aligns with our clinical findings, in which MI oocytes contributed to embryo development and, in some cases, resulted in pregnancy. However, previous studies have also reported increased rates of multinucleation and abnormal cleavage patterns in embryos derived from MI oocytes, suggesting that their developmental competence may remain compromised despite successful fertilization [18]. In this study, meiotic spindle presence in MI oocytes resistant to rescue IVM was associated with higher fertilization rates, improved embryo quality, and better pregnancy outcomes; however, these differences did not reach statistical significance.

Although all transferred embryos were morphologically Grade I, pregnancy occurred only when the embryo was derived from a spindle-positive oocyte. This finding suggests that embryo morphology alone may not fully reflect developmental competence, and meiotic spindle presence may represent an upstream marker influencing subsequent embryo quality and implantation potential. Recent evidence also suggests that early cleavage abnormalities such as multinucleation may reflect underlying defects in chromosomal segregation and developmental competence, even in morphologically high-grade embryos [19].

Spindle-positive MI oocytes may possess greater developmental competence due to more advanced nuclear organization, whereas spindle-negative oocytes likely have limited maturation progression. Therefore, polarized light imaging may represent a potentially useful, non-invasive tool to help discriminate the developmental potential of MI oocytes that fail to respond to rescue IVM, rather than discarding them uniformly.

In what concern the relationship between meiotic spindle presence and adding AOA; in the AOA-treated group, fertilization occurred more frequently in the spindle-present group compared to the spindle-absent group (71.4% vs. 50.0%). While this difference was not statistically significant, the consistent numerical difference may suggest potential biological relevance. Importantly, all cryopreserved Grade I embryos originated from spindle-positive oocytes, and both biochemical and clinical pregnancies were observed exclusively in this group. Also, in the non-AOA control group, fertilization was observed in only one patient, and this occurred in the spindle-positive subgroup, while no fertilization was detected in spindle-negative oocytes. These findings suggest that meiotic spindle presence may reflect a more advanced stage of nuclear maturation and could be associated with successful fertilization, even in the absence of artificial oocyte activation. Furthermore, the marked increase in fertilization rates observed in the AOA group indicates that artificial activation may, at least partially, compensate for deficiencies in cytoplasmic maturation in MI oocytes.

Taken together, these results support the hypothesis that both intrinsic oocyte maturity, as indicated by spindle visibility, and extrinsic stimulation through AOA may play complementary roles in enabling fertilization of MI oocytes. While spindle presence may identify oocytes with inherent developmental competence, AOA appears to be associated with enhanced fertilization potential, particularly in oocytes that might otherwise fail to activate. Overall, our findings suggest that a proportion of MI oocytes may not be irreversibly immature and, with appropriate laboratory support, can yield embryos with developmental potential. The observed pregnancy and live birth rates in this cohort should be interpreted cautiously due to the limited sample size.

In vivo-matured oocytes remain the optimal choice. For patients with a history of a low proportion of mature oocytes, several strategies can be employed to increase the number of in vivo-matured oocytes. These include adding LH to COH protocols, using a double trigger with GnRH agonist and hCG, extending the interval between hCG administration and oocyte retrieval, modifying the IVF protocol, or changing the FSH type (recombinant or hMG). However, these considerations are beyond the scope of this study.

Rescue IVM has been described using different approaches in the literature, including both prolonged culture of immature oocytes and the direct use of MI oocytes after cumulus removal. In our study, the prolonged culture method was applied, which remains a subject of debate regarding its efficiency compared with direct MI injection. Nevertheless, our findings suggest a potential role of assisted oocyte activation in supporting fertilization and embryo development from immature oocytes. The inclusion of a non-AOA control group in our study provides additional insight into the role of artificial activation. While fertilization was observed in only one oocyte in the non-AOA group, this embryo arrested at the cleavage stage and did not result in transfer. In contrast, AOA-treated oocytes demonstrated higher fertilization rates and the ability to reach transferable embryo stages. These findings suggest that AOA may be associated not only with increased fertilization but also with the ability of a subset of MI oocytes to progress beyond early developmental arrest.

One of the innovative aspects of the present study is the use of artificial oocyte activation following ICSI in MI oocytes that resisted rescue in vitro maturation. Under physiological conditions, fertilization triggers repetitive intracellular calcium oscillations mediated by sperm-derived phospholipase C zeta (PLCζ), which initiate oocyte activation, resumption of meiosis, cortical granule exocytosis, and pronuclear formation [20, 21]. In immature MI oocytes, cytoplasmic maturation may be incomplete, potentially leading to insufficient calcium signaling and suboptimal activation responses. Artificial oocyte activation aims to mimic or amplify calcium oscillations, thereby facilitating meiotic progression and early embryonic development [22].

The developmental competence of MI oocytes is generally lower than that of mature MII oocytes, primarily due to incomplete nuclear and cytoplasmic maturation. However, MI oocytes retain the molecular machinery necessary for meiotic completion, including spindle assembly components and cell-cycle regulatory proteins [23]. Following ICSI, the mechanical introduction of spermatozoa may stimulate partial activation and, when combined with artificial activation, may promote extrusion of the first polar body and transition toward a fertilization-competent state [24]. This may explain why a subset of MI oocytes can achieve fertilization and develop into morphologically high-grade embryos.

Although performing ICSI on immature MI oocytes is not conventional practice, several studies have demonstrated that MI oocytes can complete meiosis in vitro and yield viable embryos, albeit at reduced efficiency compared with MII oocytes [25, 26]. The ability of MI oocytes to be fertilized likely depends on their intrinsic maturation stage at the time of ICSI. Oocytes at late MI may be closer to the metaphase II transition and therefore more responsive to activation stimuli.

Taken together, artificial oocyte activation may help compensate for deficient endogenous activation signaling in MI oocytes and may enhance their developmental potential. However, given the exploratory nature of this approach, further large-scale studies are required to determine its safety and long-term clinical efficacy.

This study has several limitations. The first limitation is the small sample size, which restricts the generalizability of the findings. Further studies with larger sample sizes are necessary to validate these results. The second limitation is selection bias in the study population. Only couples who did not have any embryos derived from in vivo- or in vitro-matured MII oocytes received an MI-derived embryo. This indicates that the investigated population had some degree of oocyte maturation defect or a low oocyte quality profile, limiting the universality of the findings. Another limitation is that none of the embryo transfers in this study were day 5 blastocyst transfers, and no preimplantation genetic diagnosis (PGD) was performed. As a result, this study cannot provide data on blastulation rates or euploidy rates. These aspects should be addressed in future research. In addition, the developmental competence and safety of MI oocytes that fail to reach MII after short-term culture remain uncertain. The potential risk of transmitting aneuploidies or epigenetic alterations should be carefully considered before suggesting any clinical application. Therefore, the present study does not support immediate clinical use of such oocytes but rather highlights their potential and the need for further prospective research. A further limitation of this study is the relatively high rate of abnormal fertilization (e.g., 3PN/4PN), which reduces the proportion of clinically usable embryos and may be related to non-physiological activation processes associated with artificial oocyte activation. Larger studies with structured designs, appropriate control groups, and detailed chromosomal and epigenetic analyses are warranted before clinical translation can be considered. Also, the lack of statistical significance is likely attributable to the limited sample size. Larger prospective studies are needed to determine whether meiotic spindle visualization constitutes an independent predictor of reproductive outcomes in MI oocytes.

## Conclusion

This study suggests that MI oocytes that failed to mature after rescue IVM may retain limited developmental potential. The use of these MI oocytes could increase the number of transferable embryos in selected cases; however, their impact on overall clinical outcomes remains uncertain. Moreover, polarized light microscopy demonstrated that some MI oocytes exhibit a centrally positioned meiotic spindle even without polar body extrusion, indicating progression toward nuclear maturation. These observations suggest that MI oocytes are not a homogeneous group and that a subset may possess residual developmental competence. Therefore, spindle assessment may represent a potentially valuable non-invasive adjunct for identifying MI oocytes with clinical potential, although further studies are required to confirm its utility.

## Supplementary Information

Below is the link to the electronic supplementary material.

Supplementary file1 (PDF 4 KB)

Supplementary file2 (PDF 3 KB)

## Data Availability

Raw data can be shared and will be available upon request.

## Declarations

### Capsule

MI oocytes that fail to mature after rescue IVM may contribute to the number of transferable embryos and may provide additional clinical benefit in ICSI cycles.

### Attestation statement

The subjects in this trial have not concomitantly been involved in other randomized trials. Data regarding any of the subjects in the study have not been previously published. Data will be made available to the editors of the journal for review or query upon request.
