# Supplementary material for: Exploring the potential of metaphase I oocytes unresponsive to rescue IVM: fertilization, embryo development, and pregnancy outcomes
Source: Arch Gynecol Obstet. 2026 Jul 18;313(1):290. doi: 10.1007/s00404-026-08484-2 (PMC13661731; doi:10.1007/s00404-026-08484-2)
Supplement: Supplementary file 1 [file 404_2026_8484_MOESM1_ESM.pdf]

Supplementary Figure 1. Patient Flow Diagram

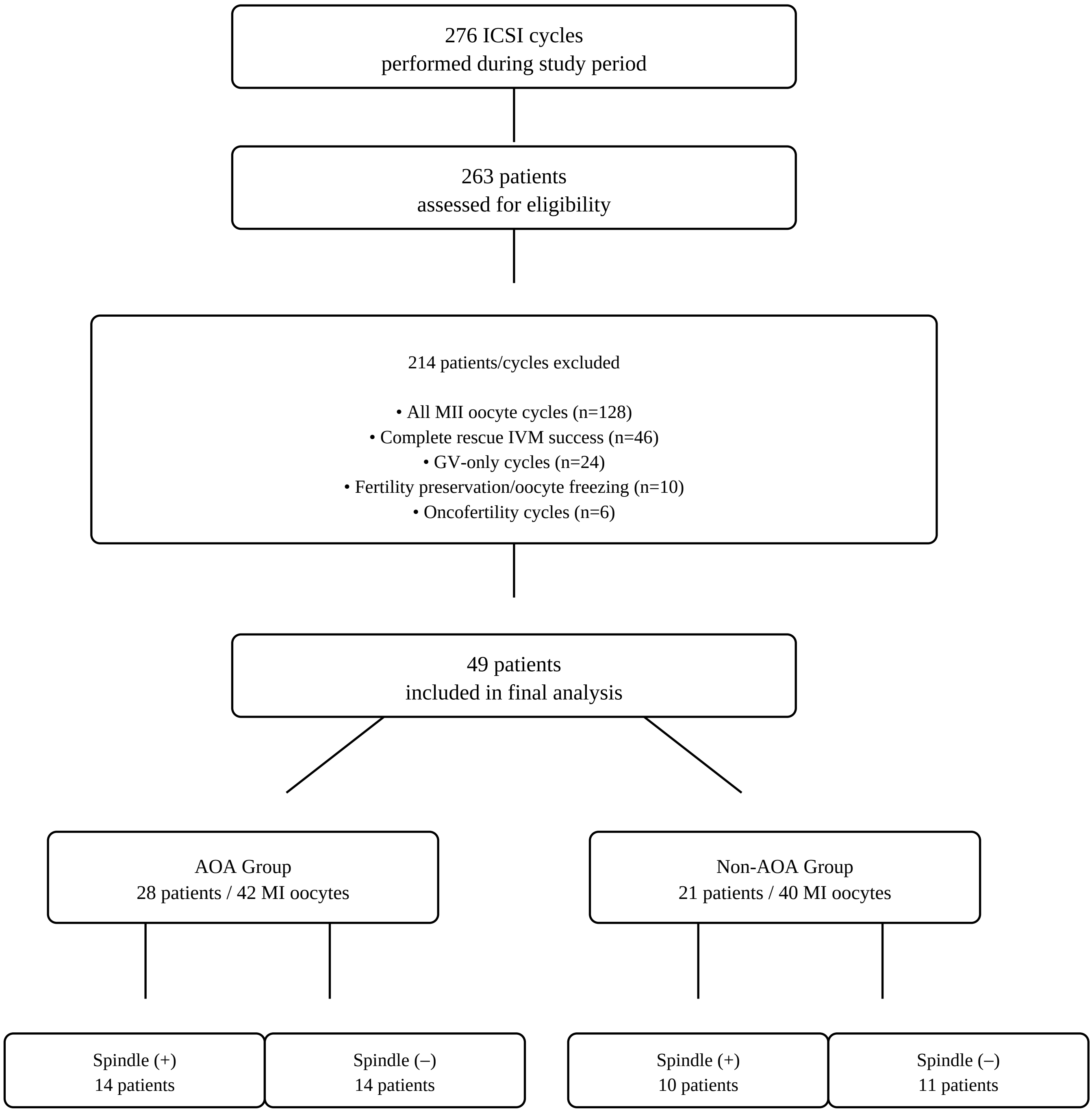

Caption: A total of 276 ICSI cycles were performed during the study period. After eligibility assessment, 214 patients/cycles were excluded according to predefined criteria, and 49 patients were included in the final analysis. The study population was divided into AOA and non-AOA groups and further stratified according to meiotic spindle status.
