# Supplementary material for: Exploring the potential of metaphase I oocytes unresponsive to rescue IVM: fertilization, embryo development, and pregnancy outcomes
Source: Arch Gynecol Obstet. 2026 Jul 18;313(1):290. doi: 10.1007/s00404-026-08484-2 (PMC13661731; doi:10.1007/s00404-026-08484-2)
Supplement: Supplementary file 2 [file 404_2026_8484_MOESM2_ESM.pdf]

Supplementary File 1. STROBE Checklist

STROBE Item	Recommendation	Reported in Manuscript
Title and Abstract	Indicate study design in title/abstract	Title, Abstract
Background/Rationale	Explain scientific background	Introduction
Objectives	State objectives/hypotheses	Introduction
Study Design	Present key elements of study design	Materials and Methods
Setting	Describe setting, locations, dates	Study Description
Participants	Eligibility criteria and selection	Inclusion/Exclusion Criteria
Variables	Define outcomes and predictors	Outcome Measures
Data Sources/Measurement	Methods of assessment	Materials and Methods
Bias	Address potential bias	Discussion – Limitations
Study Size	Explain study size	Results / Limitations
Quantitative Variables	Handling of variables	Statistical Analysis
Statistical Methods	Statistical methods used	Statistical Analysis
Participants	Numbers at each stage	Results
Descriptive Data	Characteristics of participants	Table 1
Outcome Data	Report outcome events	Tables 2–4
Main Results	Estimates and precision	Results
Other Analyses	Subgroup/exploratory analyses	Results
Key Results	Summarize findings	Discussion
Limitations	Discuss limitations	Discussion
Interpretation	Interpretation of results	Discussion
Generalizability	External validity	Discussion
Funding	Funding source	Funding Statement

This checklist summarizes compliance with the STROBE (Strengthening the Reporting of Observational Studies in Epidemiology) guidelines for reporting prospective cohort studies.
